# FKBP5-associated miRNA signature as a putative biomarker for PTSD in recently traumatized individuals

**DOI:** 10.1038/s41598-020-60334-6

**Published:** 2020-02-25

**Authors:** Hyo Jung Kang, Sujung Yoon, Suji Lee, Koeul Choi, Sihwan Seol, Shinwon Park, Eun Namgung, Tammy D. Kim, Yong-An Chung, Jungyoon Kim, Jung-Soo Han, In Kyoon Lyoo

**Affiliations:** 1 Department of Life Science, Chung-Ang University, Seoul, South Korea; 2 Ewha Brain Institute, Ewha W. University, Seoul, South Korea; 3 Department of Brain and Cognitive Sciences, Ewha W. University, Seoul, South Korea; 4 Department of Radiology, Incheon St. Mary’s Hospital, College of Medicine, The Catholic University of Korea, Seoul, South Korea; 5 Department of Biological Sciences, Konkuk University, Seoul, South Korea; 6 Graduate School of Pharmaceutical Sciences, Ewha W. University, Seoul, South Korea; 7 The Brain Institute and Department of Psychiatry, University of Utah, Salt Lake City, Utah USA

**Keywords:** Diagnostic markers, Prognostic markers, Neuroscience, Systems biology

## Abstract

The epigenetic regulation of microRNA (miRNA) expression related to the FK506-binding protein 5 (FKBP5) gene may contribute to the risk of stress-related disorders such as posttraumatic stress disorder (PTSD). Here, we identified candidate miRNAs derived from FKBP5 knockout mice as a potential diagnostic biomarker of PTSD. Using a translational approach, candidate miRNAs found to alter in expression within the medial prefrontal cortex of FKBP5 knockout mice were selected. Each candidate miRNA was examined in the serum of 48 recently traumatized individuals with PTSD and 47 healthy individuals. Multimodal imaging was also conducted to identify the neural correlates for the expression of candidate exosomal miRNAs in response to trauma exposure. Differential miRNA expression was found according to PTSD diagnosis in two composite marker groups. The differential miRNA expression between the composite marker groups contributed to PTSD symptom severity, which may be explained by differential recruitment of prefrontolimbic activity in brain imaging. The present study reveals that a set of circulating exosomal miRNAs showing altered expression in FKBP5 knockout mice play a potential role as epigenetic markers of PTSD. The corroborative evidence from multiple levels including molecular, brain, and behavioral indicates that these epigenetic biomarkers may serve as complementary measures for the diagnosis and prognosis prediction of PTSD in recently traumatized individuals.

## Introduction

Posttraumatic stress disorder (PTSD) is a psychiatric condition that results from trauma, and its pathology influences various biological processes within the body^1,2^. Emerging evidence suggests the contribution of epigenetic regulation of microRNA (miRNA) expression in the risk and protective factors associated with PTSD^1,3^, including recent reports of altered miRNA expression in response to an array of environmental stressors^4,5^. This suggests that changes in miRNA expression following trauma may serve as a promising diagnostic biomarker as well as a molecular target for mechanism-based treatments^1,3^. Given the multilevel regulatory role of miRNAs in the brain genomic response to environmental stress^5^, understanding the concerted actions of various miRNAs in relation to target gene pathways may provide insight with regards to the phenotypic heterogeneity of PTSD^6–10^. However, discrepancies exist among previous studies regarding the identification of miRNAs that are related to the pathophysiology of PTSD^1,11^. Furthermore, despite the immediate neuroplastic effects as modulated by miRNA following traumatic exposure^5,12,13^, little is known about the dynamic changes that occur in miRNA expression profiles during the acute stages of PTSD. Known for its fundamental regulatory role of the hypothalamic-pituitary-adrenal (HPA) axis and glucocorticoid receptor (GR) function in the presence of stress^14,15^, FK506-binding protein 5 (FKBP5) is a key modulator of stress-related disorders including PTSD^14,16,17^. Since the landmark study on the genetic association of FKBP5 with PTSD^18^, several preclinical and clinical studies examined the genetic modulation as well as peripheral and central expression of FKBP5 in stress-related disorders including PTSD and major depression^16,19,20^. Studies also revealed that having the risk genotype of FKBP5 may affect the prefrontolimbic circuit of the brain, an area known to regulate stress response^21–25^. Taken together, FKBP5 overexpression may be precipitated by both genetic predisposition and additional epigenetic modulation, and may therefore increase the risk for stress-related disorders with various phenotypes^8,16,26–28^.

The present study identified clusters of circulating miRNA according to their alteration in expression level in recently traumatized individuals diagnosed with PTSD, and examined the mediating role of this stress-induced epigenetic modification on the prefrontolimbic circuit of the brain. Using a translational approach, candidate miRNAs were derived from an FKBP5 knockout (KO) mouse model^29^, then examined in the human serum samples. We hypothesized that these candidate miRNA levels alter in the case of recently traumatized individuals with PTSD as compared to trauma-unexposed healthy individuals, and that these alterations in miRNA levels are associated with the structure and functional activity of the prefrontolimbic regions of the brain.

## Results

### Candidate miRNAs identified from the FKBP5 KO mouse model

The medial prefrontal expression profiles of miRNAs were compared between FKBP5 KO and FKBP5 wild-type (WT) mice. In total, 23 downregulated and 18 upregulated miRNAs were revealed in FKBP5 KO mice compared with FKBP5 WT mice (Table S1). Conservation analysis revealed that 18 out of the 41 downregulated or upregulated mouse miRNAs have cross-species sequence similarity with human miRNAs and therefore considered highly evolutionarily conserved in humans (Fig. S1 and Table S2). These miRNAs were then selected as the final candidate miRNAs for further analyses in the human PTSD cohort.

The exploratory analyses to examine the relationships between the expression profiles for the subset of identified miRNAs in the two compartments of serum exosomes and medial prefrontal cortex (mPFC) were performed and are presented in Fig. S2 and Supplementary Results.

### Clustering of candidate miRNAs and its expression profiles in PTSD

Among the aforementioned 18 candidate miRNAs selected from the FKBP5 KO mouse model, 3 of the circulating exosomal miRNAs displayed levels below the detection limit in more than 10% of the human serum samples (27, 31, and 57 unsatisfactory samples for miR-154-5p, miR-219a-5p, and miR-3074-5p, respectively), and were excluded from the final analysis.

**Figure 1:**
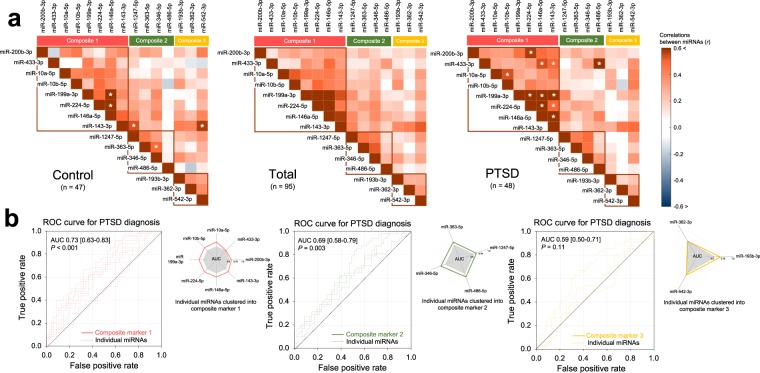
Clustering of miRNA candidates and evaluation of each resulting cluster in predicting PTSD diagnosis. (**a**) Using a principal component analysis, three composite markers were derived in a data-driven manner, consisting of highly correlated miRNAs showing a similar expression profile within each subset. Asterisks (*) in the correlation matrices of the PTSD (right panel) and control (left panel) groups indicate significant correlations at Bonferroni-corrected *P* < 0.05. (**b**) A standardized expression value of each composite marker calculated by averaging the z scores of the relative expression values of the circulating exosomal miRNAs. Diagnostic performance of each composite marker to discriminate recently traumatized individuals with PTSD from trauma-unexposed healthy individuals was evaluated with the ROC curve analysis. AUC measures indicate the discriminatory power of each composite marker, which was internally validated using 1,000 bootstrap resampling, with AUC measures of individual miRNAs within each composite marker presented in radar charts. *miRNA* microRNA, *PTSD* posttraumatic stress disorder, *ROC* receiver operating characteristic, *AUC* area under the ROC curve.

Implementing a dimension-reduction approach^30^, the principal component analysis based on a correlation matrix between the 15 candidate miRNA expression values identified three distinct clusters of ‘composite marker 1’, ‘composite marker 2’, and ‘composite marker 3’, each of which comprised of 8 miRNAs (miR-200b-3p, miR-433-3p, miR-10a-5p, miR-10b-5p, miR-199a-3p, miR-224-5p, miR-146a-5p, and miR-143-3p), 4 miRNAs (miR-1247-5p, miR-363-5p, miR-346-5p, and miR-486-5p), and 3 miRNAs (miR-193b-3p, miR-362-3p, and miR-542-3p), respectively (Fig. 1a).

The miRNA expression profiles of the PTSD group showed greater covariance pattern and higher correlation coefficients compared to the control group (Fig. 1a).

The means of the standardized miRNA expression values were compared between the two groups. The PTSD group displayed higher expression of composite marker 1 (*z* = 3.71, *P* < 0.001) and composite marker 2 (*z* = 3.01, *P* = 0.003) as compared with the control group. However, there were no between-group differences in composite marker 3 (*z* = 1.59, *P* = 0.11). Fold changes of the PTSD group in each individual miRNA are described in Fig. S3. The area under the receiver operator characteristic (ROC) curves (AUCs) calculated to evaluate the accuracy of each composite marker in diagnosing PTSD in composite markers 1 and 2 were 0.73 (95% confidence interval [CI], 0.63–0.83) and 0.69 (95% CI, 0.58–0.79), respectively. In contrast, the AUC for composite marker 3 was 0.59 (95% CI, 0.50–0.71), and was unable to differentiate between the two groups better than chance. The ROC curves for the 3 composite markers and the respective AUCs of the individual miRNAs are presented in Fig. 1b.

Detailed information on the canonical pathway of the target genes modulated by the current candidate miRNAs and the description of the theoretical bioinformatic analysis are presented in Supplementary Results

### Correlations between serum markers reflecting HPA axis and miRNA expression patterns in PTSD

**Figure 2:**
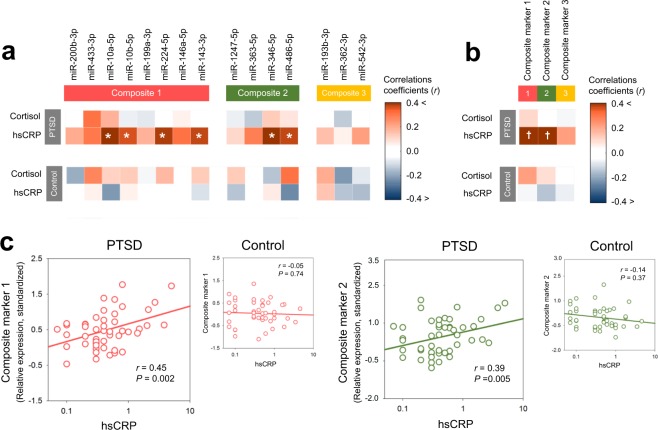
Correlations between serum markers reflecting HPA axis activity and candidate miRNA expression. (**a**) Correlation matrices between the relative expression values of individual circulating exosomal miRNAs and serum markers including cortisol and hsCRP for both PTSD and control groups. Asterisks (^*^) indicate significant correlations at *P* < 0.05. (**b**) Correlation matrices between standardized expression values of composite markers and serum markers including cortisol and hsCRP for both PTSD and control groups. Crosses (^†^) indicate significant correlations at permutation-adjusted *P* < 0.01. (**c**) Scatterplots and regression lines between hsCRP levels and standardized values of miRNA expression for composite markers 1 and 2 according to groups. Correlation coefficients were calculated using the Pearson correlation analysis. *P* values in the scatter plots indicate 5,000 permutation-adjusted values. *HPA* hypothalamus-pituitary axis, *miRNA* microRNA, *PTSD* posttraumatic stress disorder, *hsCRP* high sensitivity C-reactive protein.

There were no differences in serum levels of high-sensitivity C-reactive protein (hsCRP) (*t* = −0.14, *P* = 0.89) and cortisol (*t* = 0.62, *P* = 0.53) between the PTSD and control groups (Table 1). Serum levels of hsCRP were positively associated with composite marker 1 (*r* = 0.45, permutation-adjusted *P* = 0.002) and composite marker 2 (*r* = 0.39, permutation-adjusted *P* = 0.005) in the PTSD group, but not in the control group (composite marker 1, *r* = −0.05, permutation-adjusted *P* = 0.74; composite marker 2, *r* = −0.14, permutation-adjusted *P* = 0.37) (Fig. 2). There were no associations between serum cortisol levels and composite markers in both the PTSD (composite marker 1, *r* = 0.10, permutation-adjusted *P* = 0.49; composite marker 2, *r* = −0.01, permutation-adjusted *P* = 0.97) and control groups (composite marker 1, *r* = 0.22, permutation-adjusted *P* = 0.14; composite marker 2, *r* = 0.09, permutation-adjusted *P* = 0.55) (Fig. 2).

**Table 1:** Characteristics of recently traumatized individuals with PTSD and trauma-unexposed healthy individuals.

Characteristics	PTSD (n = 48)	Controls (n = 47)	*P* values^a^
***Demographic characteristics***			
Age, mean (SD), years	33.1 (8.6)	32.7 (8.3)	0.81
Female sex, No (%)	38 (79.2)	36 (76.6)	0.76
East Asian, No (%)	48 (100)	47 (100)	—
Right handedness, No (%)	45 (93.8)	46 (97.9)	0.62
***Trauma-related characteristics***			
Age at trauma, mean (SD), year	32.9 (9.2)	NA	—
Time since trauma, median (range), months	4.4 (1.2 to 17.6)	NA	—
**Exposed trauma type**, **No (%)**			
Physical violence	25 (52.1)	NA	—
Sexual violence	23 (47.9)	NA	—
CAPS total scores, mean (SD)	40.2 (8.1)	NA	—
***Serum markers***			
Cortisol, mean (SD), nmol/L^b^	397 (166)	419 (182)	0.53
hsCRP, mean (SD), nmol/L^c^	6.94 (9.08)	6.68 (8.60)	0.89

*SD* standard deviation, *PTSD* posttraumatic stress disorder, *No* number, *CAPS* Clinician-Administered Posttraumatic Stress Disorder Scale for DSM-5, *hsCRP* high sensitivity C-reactive protein, *NA* not applicable.

^a^*P* values were calculated using independent t-test for continuous variables and chi-square test for categorical variables.

^b^Data were not available in 1 healthy individual.

^c^Data were not available in 3 healthy individuals.

### Neural correlates of candidate miRNA expression in PTSD

**Figure 3:**
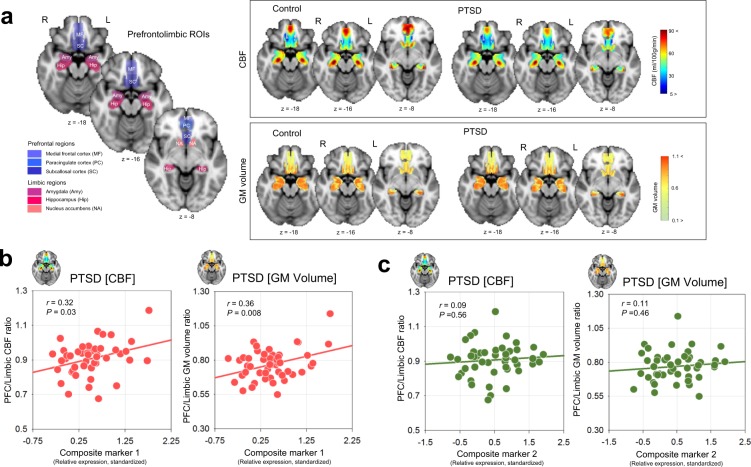
Neural correlates of candidate miRNA expression profile in PTSD. (**a**) Anatomical localization of the prefrontolimbic regions that were predefined as ROIs in this study. Mean CBF (upper panel) and GM volumes (lower panel) in the prefrontolimbic ROIs for each PTSD and control group are overlaid on the axial planes of the standard Montreal Neurological Institute template. (**b**) Scatterplots and regression lines to show the association of standardized values of miRNA expression for composite marker 1 with the ratio of CBF and GM volume between prefrontal and limbic regions of the brain, respectively. (**c**) Scatterplots and regression lines to show the association of standardized values of miRNA expression for composite marker 2 with the ratio of CBF and GM volume between prefrontal and limbic regions of the brain, respectively. *P* values in the scatter plots indicate 5,000 permutation-adjusted values. *miRNA* microRNA, *PTSD* posttraumatic stress disorder, *ROIs* regions-of-interest, *CBF* cerebral blood flow, *GM* gray matter, *L* left, *R* right.

The derived cerebral blood flow (CBF) ratio and the gray matter (GM) volume ratio between prefrontal versus limbic regions were measured to approximate the functional and structural prefrontal control over the limbic regions^31,32^, respectively, within the *a priori* defined prefrontolimbic regions-of-interests (ROIs) (Fig. 3a). The PTSD group showed reduced prefrontal/limbic CBF ratio as compared with the control group (*t* = 2.35, *P* = 0.02), while there was no between-group difference in GM volume ratio (*t* = −0.67, *P* = 0.51) (Fig. S4).

Results from the correlation analyses showed that enhanced miRNA expression levels of composite marker 1 were associated with higher prefrontal/limbic CBF ratio (*r* = 0.32, permutation-adjusted *P* = 0.03) as well as a higher GM volume ratio (*r* = 0.36, permutation-adjusted *P* = 0.008) in the PTSD group (Fig. 3b). However, these associations were not found between the miRNA expression levels of composite marker 2 and prefrontal/limbic CBF ratio (*r* = 0.09, permutation-adjusted *P* = 0.56) nor GM volume ratio (*r* = 0.11, permutation-adjusted *P* = 0.46) in the PTSD group (Fig. 3c). There were no significant correlations between miRNA expression levels in composite markers 1 or 2 and the prefrontal/limbic CBF or GM volume ratio in the control group. Correlation analyses were also performed between composite markers 1 or 2 and GM volume or CBF of individual ROI areas as to provide further supportive findings. The results from these exploratory analyses are presented in Supplementary Results.

In addition, robust regression analyses were performed to examine whether the correlations between the expression profiles of candidate miRNAs belonging to composite 1 or 2 and the prefrontal/limbic GM volume or CBF ratio differ according to trauma types. These exploratory analyses showed no interaction effects between the expression levels of composite 1 and trauma type on both the prefrontal/limbic GM volume ratio (*t* = 0.01, *P* = 0.99) or the prefrontal/limbic CBF ratio (*t* = 1.93, *P* = 0.06). Moreover, there were no significant interaction effects between the expression levels of composite 2 and trauma type on both the prefrontal/limbic GM volume ratio (*t* = 0.31, *P* = 0.76) or the prefrontal/limbic CBF ratio (*t* = −0.39, *P* = 0.70).

### Clinical implications of candidate miRNA expression in PTSD

Given the diverging roles of composite markers 1 and 2 in the prefrontal control over the limbic regions, we explored whether the imbalance between the miRNA expression levels of composite markers 1 and 2 may be associated with PTSD symptom severity. In the current study, measures of expression imbalance between composite markers 1 and 2 were calculated by subtracting the standardized expression values of composite marker 1 from those of composite marker 2.

In the PTSD group, differences between the relative expression of composite markers 1 and 2 were positively associated with PTSD symptom severity measured using the Clinician-Administered Posttraumatic Stress Disorder Scale for DSM-5 (CAPS)^33^ (*r* = 0.31, permutation-adjusted *P* = 0.03), indicating a significant relationship between the predominant expression of composite marker 2 in relation to higher severity of PTSD symptoms (Fig. S5).

## Discussion

To our knowledge, this is the first study to identify the concerted actions of a set of miRNAs showing the differential expression in FKBP5 KO mice on the pathophysiology of PTSD at the molecular, brain, and behavioral levels using a human cohort, as illustrated in Fig. S5. The primary finding of the present study is that the expression profiles of circulating exosomal miRNA candidates selected from the FKBP5 KO mouse model significantly alter in recently traumatized individuals with PTSD as compared with trauma-unexposed healthy individuals. In addition, each cluster of candidate miRNAs derived from a dimension-reduction approach showed differential effects on the prefrontolimbic structural and functional changes in the PTSD group.

One of the strengths of our translational approach of using an FKBP5 KO mouse model is the allowance to identify candidate miRNAs in a more targeted manner as compared with whole-genome miRNA expression profiling. Our findings may also have merit through having collected a comprehensive dataset from a relatively large sample size of recently traumatized individuals, where each participant underwent a battery of assessments including miRNA quantification, multimodal neuroimaging, and clinical/behavioral assessments on PTSD symptoms.

It is important to note that the majority of the currently identified miRNAs associated with PTSD have been previously implicated to take part in the underlying neural mechanisms of stress regulation and stress-related disorders (Table S3). Our findings may provide supportive evidence for previous literature on the identification of miRNAs related with stress, for which a detailed literature review is provided in Table S3 and Supplementary Results.

Consistent with a growing number of studies that have reported the involvement of FKBP5-associated genetic or epigenetic regulation in PTSD^16,19^, our proof-of-concept study found that altered expression levels of a series of candidate miRNAs derived from FKBP5 KO mice may relate to the compensatory or pathological adaptations of the prefrontolimbic circuit, a major neural correlate of PTSD. Specifically, the enhanced miRNA expression levels of composite marker 1 were associated with the increased prefrontal control over the limbic activity in the PTSD group. As this relationship was unique and exclusively applicable to miRNAs of composite marker 1 and not of composite marker 2, this may suggest a coping or compensatory role of miRNAs clustered into composite marker 1 in response to trauma exposure. While considering this potential role of miRNAs of composite marker 1, it is also noteworthy that the elapsed time since trauma exposure was relatively short in the current PTSD sample (median 4.4 months). As such, the miRNA expression profiles of the current sample may be more dynamic or differ in the case of chronic PTSD. Future studies that investigate the miRNA expression profiles of chronic PTSD may provide further insight with regards to the potential roles of each composite marker groups in the pathophysiology of PTSD.

The diverging functions between miRNAs categorized into composite markers 1 and 2 may be corroborated by our findings from the correlation analyses, which demonstrated that the imbalance in composite marker expression levels may influence PTSD symptom severity. While the evidence suggesting the direct effects of the current candidate miRNAs on FKBP5 protein expression levels within the prefrontolimbic regions is scarce, we cautiously suggest that miRNAs clustered into composite markers 1 and 2 may play a distinct role in PTSD patients through their response to altered FKBP5 protein expression in PTSD condition. However, it is also noteworthy that alteration in miRNA levels upon the ablation of FKBP5 gene may not necessarily indicate alterations in FKBP5 protein expression or activity. As such, future mechanism studies that examine the relationship between alterations in the current miRNA candidate levels and the resultant changes in FKBP5 protein expression would be necessary to support this speculation.

Among the serum markers evaluated in the current study that reflect HPA axis activity, the peripheral levels of hsCRP were significantly correlated with FKBP5-associated miRNA expression profiles in the PTSD group. This finding may imply a potential feedback link between alteration in HPA axis function and the resultant modulation of FKBP5 expression or activity, as suggested in previous research^14,16,17^. It should also be noted that the significant correlation between serum levels of hsCRP and the expression profile of the candidate miRNAs as shown in the PTSD group was not observed in the control group. Although there was no between-group difference in serum hsCRP level, this lack of correlation may be cautiously explained by previous literature, which suggested HPA axis activity is altered in individuals with PTSD^34^. Considering that HPA axis activity is partly reflected by serum hsCRP levels, the association between serum hsCRP and candidate miRNA expression profiles in the PTSD group may differ from those in the control group. Further studies to examine whether HPA axis activity may differentially influence miRNA expression profiles between being under a stressful versus normal condition would be warranted.

There were also no significant relationships between serum cortisol levels, the other marker for HPA axis activity, and candidate miRNA expression profiles in the PTSD group. While this finding may be contrary to our expectation, we suggest that this may be partly due to the less efficient feedback actions of cortisol as a result of increased glucocorticoid resistance, a key pathological feature of HPA axis activity related to PTSD^16,35^.

The current findings suggest that exosomal miRNAs may serve as adequate peripheral targets of PTSD diagnosis and treatment, given that miRNAs originating from the brain have been previously suggested to cross the blood-brain barrier by the transcytosis of exosomes^36^. Specifically, we anticipate that the expression profiles of the circulating exosomal miRNAs may reflect disease-specific alterations in miRNA expression within the brain in response to trauma. Based on these assumptions, the currently selected exosomal miRNAs may be valid neurobiological markers of PTSD in the case of those who have been recently exposed to trauma. In addition, the stability of expression patterns is another advantage for the application of exosomal miRNAs as potential biomarkers, given the fact that exosomes may serve as a RNase-protective vesicle that protects miRNAs from RNase-rich environments in the peripheral blood^37,38^. However, although a significant relationship between altered expression levels of exosomal miRNAs and mPFC miRNAs was suggested in our FKBP5 KO mouse model (Fig. S2), it should be noted that the candidate miRNAs showing altered expression levels observed in the human PTSD model may originate from other tissues or different brain regions. For the use of plasma exosomal miRNAs as biomarkers for detecting mPFC function-related events such as PTSD, further studies to replicate the links between the peripheral and mPFC expression levels of the current candidate miRNAs would be warranted.

The following limitations should be taken into consideration when interpreting the results. First of all, the current study implemented a less conservative approach using the uncorrected *P* value to detect group differences in mPFC miRNA expression levels, in order to select a relevant number of miRNA candidates for their translational application to the human model. Considering that the false discovery rate correction is more widely implemented in neuroscience-based research^39^, future studies should consider the implementation of a more conservative approach to correct for multiple comparisons and yield findings with greater validity regarding the differential patterns of mPFC miRNA expression related to FKBP KO. It should also be noted that the current statistical model for between-group differences in mPFC miRNA expression levels of the mouse model did not account for sex distribution as a relevant covariate partly due to a small sample size. In addition, the current study used the mouse reference genome GRCm38.p2 (released in 2014) for mapping. Future studies and re-analyses that implement up-to-date versions of the bioinformatics tools, including genome annotation, are warranted to ensure the accuracy of the findings.

Moreover, the current study selected candidate miRNAs based on the expression profile at the basal level from the FKBP5 KO mouse model. As such, potential alterations in miRNA expression that may occur as a reactive response to stress or trauma were not considered. Namely, the current set of miRNA candidates was not selected in the FKBP5 KO mouse model under a stress-induced condition.

As for the validity of the current findings, although internal validation using bootstrapping was performed, the current findings should be externally validated in an independent sample with a larger sample size. While taking a targeted approach by designating a predefined subset of candidates may be appropriate for proof-of-concept studies such as our own, our results should be replicated with a genome-wide expression profiling of miRNAs for the future application of exosomal miRNAs as biomarkers in the diagnosis of PTSD.

It should also be noted that the current study lacks information regarding the polymorphism of the FKBP5 gene in the human cohort of PTSD patients and trauma-unexposed individuals, despite the fact that such data may have allowed better characterization of the results. Future studies that include this important information of genetic data may provide stronger evidence on the specific vulnerability to clinical symptoms according to FKBP5 allele as well as the differential link between candidate miRNA levels and the prefrontolimbic activity in PTSD patients who carry the risk allele. Furthermore, the results from the current control group without any exposure to major trauma could not elucidate the exact effects of candidate miRNA expression on the conditional risk for PTSD among trauma-exposed individuals. The inclusion of an additional study group of trauma-exposed individuals who did not develop PTSD is warranted in order to examine whether the current miRNA candidates may differentially influence the development of PTSD in recently traumatized individuals. As such, the lack of a study group of trauma-exposed who have not developed PTSD is a notable limitation in the current study.

Finally, since alterations in miRNA expression levels may not necessarily reflect their effects on the target mRNA of the canonical pathway, further studies are necessary to elucidate the detailed mechanisms underlying the associations between the current miRNA candidates, their respective target mRNAs, and the overall biological processes that may be involved. The current results should be interpreted with this important limitation in mind.

In conclusion, the current proof-of-concept study suggests the possibility of utilizing exosomal miRNA as biomarkers that are complementary to predicting the prognosis of PTSD in recently traumatized individuals. Further applications using FKBP5-associated miRNAs as biomarkers may be an important step towards the development of mechanism-based, disease-modifying or preventive treatment of PTSD.

## Methods

### Selection of candidate miRNAs and examination of stress responses in the FKBP5 KO mouse model

Strains of mice (Fkbp5tm1Dvds/J) used for this study included male and female of FKBP5 knockout (KO, n = 4) and littermate wild type (WT, n = 4). FKBP5 KO mice were obtained from the Jackson Laboratory (Bar Harbor, Maine, USA). Three to four-month-old KO mice (JAX stock #017989) and WT mice were used for the study. RNA extraction was performed from the dissected mPFC using a miRNeasy mini kit (Qiagen, Hilden, Germany). Total RNA extracted from the brain was used for small RNA enrichment. Column-purified small RNA was used for library preparation, using the NEXTflex Small RNA Sequencing Kit V3 (Bioo Scientific, Austin, TX, USA). In addition to examining the miRNA expression within the mPFC, circulating miRNA expression levels were also examined in the blood of FKBP5 KO and WT mice.

Detailed procedures of the animal study are described in Supplementary Methods. All animal use and related protocols were in accordance with the Institutional Animal Care and Use Committee of Chung-Ang University and Konkuk University.

### Participants

The study recruited 50 recently traumatized individuals diagnosed with PTSD at the time of assessment (PTSD group) and 50 trauma-unexposed healthy individuals (control group), all of whom were between the ages of 20 and 50. Among the 100 participants that were enrolled, 2 individuals from the PTSD group and 3 individuals from the control group were excluded due to insufficient exosomal miRNA yield and quality. The final analysis included 48 individuals from the PTSD group and 47 individuals from the control group. The demographic and clinical characteristics of the study participants are presented in Table 1. A schematic overview of the current study design is illustrated in Fig. S1. The authors assert that all procedures contributing to this work comply with the ethical standards of the relevant national and institutional committees on human experimentation and with the Helsinki Declaration of 1975, as revised in 2008. All procedures involving human subjects were approved by the institutional review board of Ewha W. University. Written informed consent was obtained from all subjects.

### Identification of putative FKBP5-associated miRNA markers in a human PTSD cohort

Exosomal RNA was isolated from human serum using ExoQuick solution (System Biosciences, Mountain View, CA, USA). Isolated exosome RNAs were reverse-transcribed using the TaqMan MicroRNA Reverse Transcription kit (Applied Biosystems, Foster City, CA, USA) and the miRNA-specific reverse transcriptase primers. All miRNA expression values were normalized to that of miR-16 and relative expression values of miRNAs were used for subsequent statistical analyses. Further details regarding the examination of peripheral miRNA levels in a human cohort are provided in Supplementary Methods.

### Examination of serum markers reflecting HPA axis activity

Serum levels of cortisol and hsCRP were measured to reflect HPA axis activity. For each serum sample, levels of cortisol and hsCRP were examined in duplicate using commercialized kits containing reagents and automated devices, as further described in Supplementary Methods.

### Examination of neural correlates of FKBP5-associated miRNA expression in PTSD

Structural and perfusion magnetic resonance (MR) imaging scans were obtained using a 3.0 Tesla Philips Achieva MR scanner (Philips Medical System, Netherlands) equipped with a 32-channel head coil. High-resolution T1-weighted images and arterial spin labeling (ASL) images were acquired using a three-dimensional T1-weighted magnetization-prepared rapid gradient echo imaging sequence and a pseudocontinuous ASL single-shot echo-planar imaging sequence, respectively.

Absolute CBF (ml/100 g/min) and GM volumes were measured as neural correlates of FKBP5-associated miRNA expression in relation to PTSD in predefined ROIs, mainly including the prefrontolimbic regions. The prefrontal regions included the medial prefrontal cortex, paracingulate cortex, and subcallosal cortex, and the limbic regions included the amygdala, hippocampus, and nucleus accumbens.

Details regarding the image acquisition, preprocessing, selection of the prefrontolimbic ROIs, and measurements of CBF and GM volumes of the *a priori* defined ROIs are described in Supplementary Methods.

### Statistical analyses

A principal component analysis using a correlation matrix between miRNA expression levels followed by a varimax rotation was performed to extract the initial set of components as relevant composite markers for miRNAs.

Group comparisons of FKBP5-associated exosomal miRNA levels between the PTSD and control group were performed using logistic regression analysis. The AUCs for the miRNA candidates was calculated with an internal validation of 1,000 bootstrap resampling.

Pearson correlation analyses were performed between the relative expression profiles of miRNA candidates and serum cortisol and hsCRP levels, respectively. In addition, the relationships between standardized expression profiles of the miRNA composite markers and CBF as well as GM ratio of the prefrontal versus limbic regions were examined. Permutation-adjusted *P* values were calculated in order to correct for multiple comparisons^40^.

Further details regarding the statistical analyses performed are described in Supplementary Methods.

### Ethics

This study was approved by the Institutional Review Board of Ewha W. University and conforms to the Declaration of Helsinki. All participants gave written informed consent. All animal experiments and related protocols were in accordance with the guidelines approved by the Institutional Animal Care and Use Committee of Chung-Ang University and Konkuk University.

## Supplementary information

Supplementary information.

## Data Availability

The datasets analysed in the current study are available from the corresponding author on reasonable request.
