# Supplementary material for: FKBP5-associated miRNA signature as a putative biomarker for PTSD in recently traumatized individuals
Source: Sci Rep. 2020 Feb 25;10:3353. doi: 10.1038/s41598-020-60334-6 (PMC7042218; doi:10.1038/s41598-020-60334-6)
Supplement: Supplementary file 1 — Supplementary information. [file 41598_2020_60334_MOESM1_ESM.docx]

**Supplementary Information**

FKBP5-associated miRNA signature as a putative biomarker for PTSD in recently traumatized individuals

**Authors**: Hyo Jung Kang, Sujung Yoon, Suji Lee, Koeul Choi, Sihwan Seol, Shinwon Park, Eun Namgung, Tammy D. Kim, Yong-An Chung, Jungyoon Kim, Jung-Soo Han, In Kyoon Lyoo

**Corresponding Author**: In Kyoon Lyoo

**Email**: inkylyoo@ewha.ac.kr

**This Supplementary Information includes:**

Supplementary Methods

Supplementary Results

Figures S1 to S5

Tables S1 to S3

References for Supplementary Information

**Supplementary Methods**

**Participants**

In the PTSD group, individuals had recently been exposed to either of the following events within the past 18 months (median 4.4 months, range 1.2 - 17.6 months): 1) directly experiencing a life-threatening traumatic event or 2) learning of a violent death or a life-threatening accident of a closely related person. The diagnosis and symptom severity of PTSD were assessed using the Clinician Administered PTSD Scale for DSM-5 (CAPS-5)^1^. We excluded individuals with any of the following conditions: significant medical conditions; lifetime Axis 1 psychiatric disorders including PTSD (prior to the index trauma in the case of the trauma-exposed group), psychotic disorders, or bipolar disorder; Axis 2 antisocial or borderline personality disorders; a history of traumatic brain injury with loss of consciousness; contraindications to brain magnetic resonance imaging (MRI). A schematic overview of the current study design is illustrated in Figure S1.

**Selection of candidate miRNAs from the FKBP5 Knockout (KO) mouse model**

*Animal models:* Strains of mice (Fkbp5tm1Dvds/J) used for this study included male and female FKBP5 knockout (KO, n = 4) and littermate wild type (WT, n = 4). The sex distribution for the entire group was 62.5% male and 37.5% female (3 male and 1 female FKBP5 KO mice, and 2 male and 2 female WT mice). All the mice were 11 weeks old, and weighed between 20 and 30 grams. The FKBP5 KO mice were obtained from a commercial stock by Jackson Laboratory (Bar Harbor, Maine, USA)(JAX stock #017989), which were bred by crossing FKBP5 heterozygous male and heterozygous female mice. All mice were genotyped using polymerase chain reaction amplification of the genomic DNA, which was extracted from the tail tips before the experiments. Mice were housed in a temperature- and humidity-controlled room (22 ± 1°C, 50 ± 10 %) that is entrained to a light-dark cycle of 12 hours light and 12 hours darkness (lights on/off at 0800/2000 hour), and food and water were available ad libitum. An experimenter handled the mice for 6 days prior to the behavior procedure. All behavioral procedures were performed during the light phase. The mice were anesthetized with isoflurane before the collection of blood by inferior vena cava puncture. Anesthesia was used to avoid sampling-associated stress. After blood-sampling, the mice were sacrificed by cervical dislocation. Sacrificing of the mice for brain dissections were conducted between 9 AM and 12 PM as to control for stress-related factors. Upon the completion of the brain extraction, RNA extraction was performed from the medial prefrontal cortex (mPFC) using a miRNeasy mini kit (Qiagen, Hilden, Germany). The animal experiments were conducted in accordance with the Chung-Ang University and Konkuk University Council Directive for the use and care of laboratory animals, and National Institutes of Health animal care guidelines. The Institutional Animal Care and Use Committee of Chung-Ang University and Konkuk University approved all protocols described in this study.

*RNA extraction from the medial frontal cortex:* RNA extraction was performed using a miRNeasy mini kit (Qiagen, Hilden, Germany). For the total RNA, dissected tissues were homogenized using a Bullet Blender (Next advance, Averill Park, NY, USA) with QIAzol lysis reagent and RNase free beads. The homogenate was combined with chloroform and centrifuged for 15 minutes at 12 000 X g at 4°C. The supernatant was combined with 100% ethanol and applied to the spin column. Flow-through solution was purified using a RNeasy minElute cleanup kit (Qiagen, Hilden, Germany) for miRNA enrichment. Subsequent processes were performed according to the manufacturer's instructions. Optical density values of extracted RNA were measured using NanoDrop (Thermo Scientific, Wilmington, DE, USA) to confirm an A260:A280 ratio above 1.9. RNA Integrity Number was determined for each sample using a Bioanalyzer RNA 6000 Nano Kit or Bioanalyzer RNA 6000 Pico Kit (Agilent Technologies, Santa Clara, CA, USA), depending upon the total amount of RNA.

*RNA purification and small RNA sequencing:* RNA extraction was performed using miRNeasy mini kit (Qiagen, Germany). Dissected tissues were homogenized using a Bullet Blender (Next advance, USA) with QIAzol lysis reagent and RNase free stainless beads. The homogenate was combined with chloroform and centrifuges for 15 min at 12,000 g at 4°C. The supernatant was combined with 100% ethanol and applied to the spin column. Flow-through solution was purified using RNeasy minElute cleanup kit (Qiagen, Germany) for miRNA enrichment. Subsequent processes were performed according to the manufacturer’s instructions. The concentration of miRNA was quantified using NanoDrop (Thermo Scientific, USA). Small RNA samples were used for library preparation, using a NEXT flex small RNA-Seq Library Prep Kit V3 (Bioo Scientific, USA) according to manufacturer’s protocol. RNA adapters were ligated to 3’ and 5’ end of the RNA molecule and adapter-ligated RNA was reverse transcribed into single-stranded cDNA. The cDNA was then PCR amplified and sequenced using Illumina HiSeq 2500 platform (HiSeq v4 Chemistry) in 100PE mode (Illumina Inc., San Diego, CA, USA). Raw data were submitted to the GEO (Gene Expression Omnibus) repository under accession number (GSE 139502).

*Pre-processing and processing of the small RNA sequencing data:* The raw RNA sequences were processed with cutadapt (v1.11) to remove the sequencing adapters. After this step, quality control checks on raw sequence data was performed using Fast QC (v0.11.5). Low-quality bases below Q20 were trimmed from 3’ ends of each read, and reads that are shorter than 17 nucleotides were discarded. The remaining high-quality reads between 18 and 30 nucleotides were selected and mapped onto the mouse reference genome GRCm38.p2, using BowTie (v1.1.2) with only 1 mismatch allowed (-n 1). Reads showing more than 5 alignments were excluded. The reads mapped to each gene for each sample were then counted using HTseq (v0.6.1p1) with the 'intersection-nonempty' mode based on miRBase (release 20).

*Differential expression analysis:* Differentially expressed genes were performed on the identified miRNAs using edgeR in R (v3.10.2). Default parameters were used for edgeR including the trimmed mean method (TMM) normalization. Differentially expressed miRNAs were determined by *P* value of < 0.05, which was calculated by Fisher’s exact test.

*Assessment of circulating miRNA expression levels:* Blood was collected from FKBP5 KO mice and FKBP5 WT mice in serum collection tubes. The blood was incubated for 1 hour in ice before being centrifuged at 8 000 rpm for 2 minutes at room temperature. The supernatant was transferred into an Eppendorf tube and stored at -80°C. The serum was centrifuged at 3 000 X g for 15 minutes to remove cells and cell debris. Exosome isolation from mouse blood was performed as described below. After transferring supernatants into a new Eppendorf tube, exosomes were isolated by ExoQuick exosome precipitation solution (System Biosciences, Mountain View, CA, USA) according to the manufacturer's guidelines. A 1/4 volume of ExoQuick solution was added to the serum and the mixture was incubated for 30 minutes in ice. The mixture was centrifuged at 1,500 X g for 30 minutes and supernatants were removed. Residual ExoQuick solution was then spun down by centrifugation at 1,500 X g for 5 minutes. The exosome pellets were obtained after the supernatants were aspirated. Exosomal RNA was extracted from serum exosomes using the total exosome RNA and protein isolation kit (Invitrogen, Carlsbad, CA, USA) according to the manufacturer’s instructions. The expression profile for the subset of miRNAs that were differentially expressed in the mPFC of FKBP5 KO mice relative to WT mice (mmu-miR-690, mmu-miR-465c-5p, mmu-miR-1194, mmu-miR-139-3p, mmu-miR-10b-5p, mmu-miR-486-5p, mmu-miR-146a-5p), were examined using real-time PCR.

**Identification of putative FKBP5-associated miRNAs markers in a human PTSD cohort**

*Sample collection:* Serum was collected from participants in the morning between 9 AM and 11 AM. Peripheral venous blood samples were drawn from the participants and collected in a serum-separating tube. The serum sample was reserved for 30 minutes in room temperature, then centrifuged at 3,000 rpm to separate the serum. Aliquots of the serum were stored immediately after the centrifugation at a temperature of -70°C until the time of analyses.

*Exosomal RNA isolation from human serum:* The blood was incubated for 1 hour in ice before being centrifuged at 8,000 rpm for 2 minutes at room temperature. The supernatant was transferred into an Eppendorf tube and stored at -80°C. The serum was centrifuged at 3 000 X g for 15 minutes to remove cells and cell debris. After transferring supernatants into a new Eppendorf tube, exosomes were isolated from human serum using ExoQuick solution (Cat# EXOQ5A-1, System Biosciences, Mountain View, CA, USA) according to the manufacturer’s instructions. In brief, 150 μl of serum was centrifuged at 3 000 X g for 15 minutes to remove cells and debris, then 38 μl of Exoquick solution were added to each sample. Mixtures were incubated at 4°C for 30 minutes then centrifuged at 4°C for 30 minutes to precipitate exosome pellets. Exosome pellets were resuspended with an exosome resuspension buffer supplied by the exosome RNA isolation kit.

*Quantitative real-time PCR:* Isolated exosome RNAs were reverse-transcribed using the TaqMan microRNA Reverse Transcription kit (Cat# 4366459, Applied Biosystems, Foster City, CA, USA) and the miRNA-specific specific RT primers according to the manufacturer’s instruction. Twenty ng of RNA were mixed with 3 μl of specific stem-loop primer and 7 μl of reverse transcriptase (RT) master mix. Assays were performed in triplicate with 1.33 μl of cDNA mixed with qPCR master mix (Cat# 4440043, Applied Biosystems, USA). Real-time PCR was performed in 384-well plates by using the ABI QuantStudio 6 Flex Real-Time PCR system (Cat# 4485694, Applied Biosystems, Waltham, MA, USA). Thermal cycling conditions were as follows: 95°C for 10 minutes, followed by 40 cycles of 95°C for 25 seconds, then at 60°C for 1 minute (1.6°C/s ramp rate). Each PCR reaction was carried out in triplicate. All the miRNA expression value was normalized to that of miR-16. The delta Ct (ΔC_t_) values were calculated from the C_t_ values using the normalization controls (miR-16). For each miRNA, ΔΔC_t_ values were obtained by subtracting ΔC_t_ values of the PTSD group from those of the corresponding control group and these values were converted to calculate the fold change (2^-ΔΔCt^). Relative expression values of miRNAs were set as the log 2 transformation of the fold change and were used for subsequent statistical analyses.

*Prediction and enrichment analysis of target genes modulated by candidate miRNAs:* The target genes of differentially expressed miRNAs were predicted by five online software tools including miRDB (http://www.mirdb.org), miRanda (http://www.microrna.org), DIANA (http://diana.imis.athena-innovation.gr/DianaTools), miRmap (http://mirmap.ezlab.org), and TargetScan (http://www.targetscan.org). The top 50 genes were obtained in each database and the genes predicted in two or more database were sorted.

**Examination of serum markers reflecting HPA axis activity**

Serum cortisol levels were measured by a radio-immunoassay (RIA) kit (Cortisol RIA CT [AMP 80-R71200], Asbach Medical Products GmbH, Obrigheim, Germany) using the r-counter (COBRA 5010 Quantum, Packard, USA). Serum high sensitivity C-reactive protein (hsCRP) levels were measured using an immunoturbidimetric assay kit (Cardiac C-Reactive Protein [Latex] High Sensitive [ACN 8217], Diagnostica Stago, Asnieres, France) and analyzer (COBAS 8000 c702 Chemistry Autoanalyzer, Diagnostics International, Rotkreuz, Switzerland). All peripheral blood biomarker measures were acquired by personnel who were blind to the participants' information at the Green Cross Laboratories (Yongin, Gyunggi-do, South Korea), certified by the College of American Pathologists' Laboratory Accreditation Program and the German External Quality Assessment Scheme For Analyses in Biological Materials.

**Examination of neural correlates of candidate miRNA expression in PTSD**

*Image acquisition and preprocessing:* Structural and perfusion magnetic resonance imaging (MRI) scans were obtained using a 3.0 Tesla Philips Achieva magnetic resonance (MR) scanner (Philips Medical System, Netherlands) equipped with a 32-channel head coil. High-resolution T1-weighted images were acquired using a three-dimensional T1-weighted magnetization-prepared rapid gradient echo imaging sequence and the acquisition parameters were as follows: echo time (TE), 3.4 ms; repetition time (TR), 7.4 ms; flip angle, 8º; field of view (FOV), 220 X 220 mm^2^; slice thickness, 1 mm; 180 contiguous sagittal slices. Arterial spin labeling (ASL) images were acquired using a pseudocontinuous ASL (pCASL) single-shot echo-planar imaging sequence with the following parameters: TE, 11 ms; TR, 4 000 ms; FOV, 220 × 220 mm^2^; voxel size, 2.75 × 2.75 mm^2^; slice thickness, 6 mm; 18 slices; labeling duration, 1 650 ms; post labeling delay, 1 600 ms; 40 control-label pairs. The labeling plane was positioned 85 mm inferior to the center of acquisition volume perpendicular to the internal carotid arteries. For estimation of equilibrium magnetization of arterial blood, an echo-planar imaging proton density (M0) image was acquired (no labeling or background suppression; TR, 6 000 ms; all other parameters identical to the pCASL scan).

T2-weighted (TE, 80 ms; TR, 3,381 ms; flip angle, 90º; FOV, 220 X 220 mm^2^; slice thickness, 3 mm; 35 slices) and fluid-attenuated inversion recovery axial images (TE, 276 ms; TR, 4 800 ms; inverse time [TI], 1 650 ms; FOV, 240 X 240 mm^2^; slice thickness, 0.56 mm) were also acquired to screen for the presence of neuroradiological abnormalities.

*Selection of the prefrontolimbic ROIs:* Regions-of-interest (ROI) masks for the prefrontolimbic regions were generated *a priori* from the Harvard-Oxford Structural Atlas^2^. The prefrontal regions included the medial prefrontal cortex, paracingulate cortex, and subcallosal cortex, and the limbic regions included the amygdala, hippocampus, and nucleus accumbens (Figure 4A).

*Measurement of CBF of a priori defined ROIs:* Preprocessing of ASL images and quantification of absolute cerebral blood flow (CBF, ml/100g/min) was performed using FMRIB Software Library tools (FSL, http://www.fmrib.ox.ac.uk/fsl). As preprocessing steps, the control and label images were motion-corrected, pair-wise subtracted, and averaged to obtain the mean perfusion-weighted images. Quantification of absolute CBF was performed by estimation of the equilibrium magnetization of arterial blood flow using a saturation inversion recovery of the control image (M0) at voxel-level^3^. The variational Bayesian approach was implemented for this calibration step using the Bayesian Inference for Arterial Spin Labeling (BASIL) tool from the FMRIB's Software Library Toolbox (http://fsl.fmrib.ox.ac.uk/fsl/fslwiki/BASIL)^4,5^. The calibrated images were quantified into absolute CBF maps using a single compartment model described elsewhere^3^ with the following parameters: labeling efficiency of 0.85, blood-brain barrier coefficient of 0.9 mL/g, labeling duration of 1 650 ms, post labeling delay of 1 600 ms, and longitudinal relaxation time of arterial blood of 1.65 second.

Extraction of CBF values from the prefrontal and limbic regions was performed in ASL native space with the following registration steps. Individual calibrated CBF maps in ASL space were coregistered to the corresponding T1-weighted images using affine transformation. The individual T1-weighted images were nonlinearly registered to the T1 template in the Montreal Neurological Institute (MNI) space. Using the resultant transformation matrices in this two-step registration, inverse transformation was applied to warp the *a priori* defined prefrontolimbic ROIs in the MNI space to the subject-specific prefrontolimbic regions in the ASL native space. In parallel, gray matter (GM) partial volume images were created by segmenting high-resolution T1-weighted images using FSL-FAST^6^. The GM partial volume image of each individual in T1 space was registered to the corresponding calibrated cerebrospinal fluid (CSF) by applying aninverse transformation matrix, with a threshold of 0.3 to create GM masks, which were used to extract CBF values within these masks. As such, ROI values were restricted to voxels predominantly composed of predominantly GM.

*Measurement of GM volumes of a priori defined ROIs:* A voxel-based morphometric (VBM) approach was used to estimate GM volumes of the prefrontolimbic ROIs^7,8^. All procedures were processed using the FSL (http://www.fmrib.ox.ac.uk/fsl). Briefly, the non-brain tissues of T1-weighted images were removed and probabilistic tissue classification of GM, white matter, and CSF density was performed. The GM partial volume images were used to create the initial template by linear registration to the MNI standard space and then averaging the aligned images. GM images in the native space were normalized to this initial template, then averaged to create a final study-specific template. Subsequently, normalization was conducted between each GM image and the study-specific template. All registered images were modulated to estimate GM volume at each voxel and were then smoothed with an isotropic Gaussian kernel with full width at half maximum of 4.6 mm. The average GM volume of all voxels within the prefrontal as well as limbic ROI masks were calculated and used for further analyses.

**Statistical Analyses**

*Group differences in exosomal miRNA levels between the PTSD and control groups*: A principal component analysis using a correlation matrix between miRNA expression levels and a varimax rotation was performed to reduce data dimensionality and to extract the relevant set of components. Multiple imputations using multivariate normal imputation was performed to account for missing miRNA expression values^9^. The average value from 20 imputations of the missing values was used to replace the missing data points for each miRNA expression level (the number of missing data; miR-200b-3p, n = 4; miR-433-3p, n = 1; miR-10a-5p, n = 0; miR-10b-5p, n = 1; miR-199a-3p, n = 0; miR-224-5p, n = 4; miR-146a-5p, n = 1; miR-143-3p, n = 0; miR-1247-5p, n = 4; miR-363-5p, n = 7; miR-346-5p, n = 1; miR-486-5p, n = 4; miR-193b-3p, n = 3; miR-362-3p, n = 2; miR-542-3p, n = 3). Components with an eigenvalue greater than 1.3 were retained to generate a three component solution.

Relative expression levels of miRNA were standardized to z scores using the mean and standard deviation values of the control group. The z scores of relative expression levels of the miRNAs comprised in each composite marker were averaged to construct a standardized relative expression level for each composite marker.

Group differences in FKBP5-associated candidate miRNAs as well as composite markers were analyzed using logistic regression analysis. Sensitivity analyses were performed to include additional covariates including age and sex composition. The area under the ROC curve (AUC) for miRNA candidates was calculated with an internal validation of 1 000 bootstrap resampling.

*Relationships between exosomal miRNA levels and peripheral marker:* Pearson correlation analyses were performed to examine the relationships between relative expression profiles of miRNA candidates and serum cortisol or hsCRP levels. Permutation-adjusted *P* values were calculated to correct for multiple comparisons^10^. A total of 5 000 permutations were performed to obtain an empirical null distribution of effects under the null-hypothesis. The proportion of the null distribution that was greater than the observed values from the actual data set was computed as a permutation-adjusted *P* value^10^.

*Relationships between exosomal miRNA levels and prefrontolimbic activity:* CBF ratio between the prefrontal and limbic regions were calculated as functional measures for prefrontal control over limbic regions. Similarly, the GM ratio between the prefrontal and limbic regions were calculated as structural measures for prefrontolimbic regulation. Pearson correlation analyses were performed to examine the relationships between standardized expression profiles of the miRNA composite markers and both CBF ratio and GM ratio of prefrontal/limbic regions. Permutation-adjusted *P* values were calculated in order to correct for multiple comparisons^10^.

**Supplementary Results**

**Relationships between circulating exosomal and medial prefrontal expression profiles for the subset of identified miRNAs**

Exploratory analyses were performed to investigate whether the profiles of expression between serum exosomes and mPFC are similar in nature for both FKBP5-KO and WT mice. Linear regression analysis was performed between the serum exosome and mPFC expression levels for the following 9 miRNA candidates: miR-690, miR-1194, miR-465c-5p, miR-139-3p, miR-10b-5p, miR-146a-5p, miR-486a/b-5p, miR-1934-5p, and miR-200b-5p. The correlation between serum exosome and medial prefrontal cortex expression levels were significant in the positive direction for miR-690, miR-1194, miR-465c-5p, miR-139-3p, and miR-486a/b-5p (Figure S2).

**Results from theoretical bioinformatic analysis of significant target genes predicted by candidate miRNAs**

Target gene search analysis of differentially expressed miRNAs in the PTSD group was performed in order to gain insight regarding the detailed biological processes. Based on results from the 5 aforementioned database (TargetScan, miRmap, miRDB, miRanda, and DIANA), we selected the target gene candidates which were indicated from at least 2 of the database for further gene ontology analyses. Gene ontology analyses suggested several biological processes involving the differentially expressed miRNAs in the PTSD group as follows: regulation of transcription (*P* = 3.8 X 10^-7^), regulation of synaptic transmission (*P* = 1.5 X 10^-2^), neuron differentiation (*P* = 7.6 X 10^-3^), learning and memory behavior (*P* = 1.5 X 10^-2^), Wnt signaling pathway (*P* = 4.9 X 10^-2^), Toll-like receptor signaling (*P* = 6.8 X 10^-3^), Glioma (*P* = 6.5 X 10^-5^), and endometrial cancer (*P* = 1.3 X 10^-4^).

**Relationships between candidate miRNA expression and GM volume or CBF of individual ROIs**

As exploratory analyses, correlation analyses were also performed between candidate miRNA expression levels and GM volumes or CBF of the prefrontolimbic areas. In the PTSD group, enhanced miRNA expression levels of composite marker 1 were significantly associated with the prefrontal GM volume (total prefrontal ROIs, *r* = 0.40, *P* = 0.005; medial frontal cortex ROI, *r* = -0.02, *P* = 0.91; paracingulate cortex ROI, *r* = 0.43, *P* = 0.003; subcallosal cortex ROI, *r* = 0.30, *P* = 0.04), but not with the CBF values of any prefrontal ROIs (total prefrontal ROIs, *r* = 0.11, *P* = 0.45; medial frontal cortex ROI, *r* = 0.11, *P* = 0.45; paracingulate cortex ROI, *r* = 0.09, *P* = 0.57; subcallosal cortex ROI, *r* = 0.17, *P* = 0.26). For the limbic areas, the PTSD group did not show any significant relationships between the miRNA expression levels of composite marker 1 and GM volume (total limbic ROIs, *r* = -0.12, *P* = 0.43; amygdala ROI, *r* = -0.04, *P* = 0.79; hippocampus ROI, *r* = -0.16, *P* = 0.28; nucleus accumbens ROI, *r* = 0.02, *P* = 0.90) nor CBF of the limbic areas (total limbic ROIs, *r* = -0.04, *P* = 0.80; amygdala ROI, *r* = -0.02, *P* = 0.88; hippocampus ROI, *r* = -0.06, *P* = 0.70; nucleus accumbens ROI, *r* = 0.08, *P* = 0.58). Although expression levels of composite 1 marker were not significantly associated with the prefrontal CBF partly because of its static nature^11,12^, these exploratory analyses may suggest that the potential influences of enhanced miRNA expression levels of composite marker 1 on the prefrontolimbic regions may be in part derived from its effect on the prefrontal regions.

There were no significant relationships between miRNA expression levels of composite marker 2 and the prefrontal volume (total prefrontal ROIs, *r* = 0.10, *P* = 0.50; medial frontal cortex ROI, *r* = 0.12, *P* = 0.42; paracingulate cortex ROI, *r* = 0.19, *P* = 0.20; subcallosal cortex ROI, *r* = -0.14, *P* = 0.34) or CBF (total prefrontal ROIs, *r* = 0.02, *P* = 0.88; medial frontal cortex ROI, *r* = 0.10, *P* = 0.49; paracingulate cortex ROI, *r* = -0.03, *P* = 0.85; subcallosal cortex ROI, *r* = 0.05, *P* = 0.73) in the PTSD group. In addition, the expression levels of composite maker 2 were not associated with GM volumes of the limbic regions (total limbic ROIs, *r* = -0.11, *P* = 0.48; amygdala ROI, *r* = -0.18, *P* = 0.22; hippocampus ROI, *r* = -0.03, *P* = 0.85; nucleus accumbens ROI, *r* = -0.13, *P* = 0.37) or CBF (total limbic ROIs, *r* = -0.03, *P* = 0.86; amygdala ROI, *r* = 0.03, *P* = 0.84; hippocampus ROI, *r* = -0.06, *P* = 0.71; nucleus accumbens ROI, *r* = 0.07, *P* = 0.64) in the PTSD group.

**Previous literature review regarding miRNA alterations related to PTSD observed in the current study**

As briefly summarized in Table S3, previous studies have reported the involvement of particular miRNAs in modulating stress responses, as examined in various experimental models including but not limited to repeated immobilization, restraint, tail shock, and fear conditioning models. The miRNAs found to be associated with PTSD in the current findings that also coincide with previous reports are as follows: miR-200^13-20^, miR-433^13,17^, miR-10^14,17,21^, miR-199^14,22,23^, miR-146^14^, and miR-143^15,24^ (all clustered into composite marker 1 in the current study) as well as miR-363^13,17^ and miR-486^24,25^ (clustered into composite marker 2). In these studies, altered expression levels of miRNAs were likely to influence a diverse range of behavioral patterns in response to stress, encompassing both an increase and decrease of anxiety-like behaviors^16,26^. It has also been suggested that a concerted effort by multiple miRNAs is required in the regulation of stress response, which may exert differential effects including an initial protective approach to stress followed by a pathological influence on the disease course^26^. Although the comprehensive research attempts to analyze genome-wide expression profiling are few in number and with relatively small sample sizes, differential expression levels of miR-200, miR-10, miR-199, miR-143, miR-363 and miR-486 in relation to PTSD^22,24,25^ or major depressive disorder^13,14, 23, 27^ have been previously reported in human cohort studies. Taken together, these findings suggest that FKBP5-associated miRNAs examined in the current study may be actively involved in the pathological or compensatory mechanisms underlying stress responses.

| 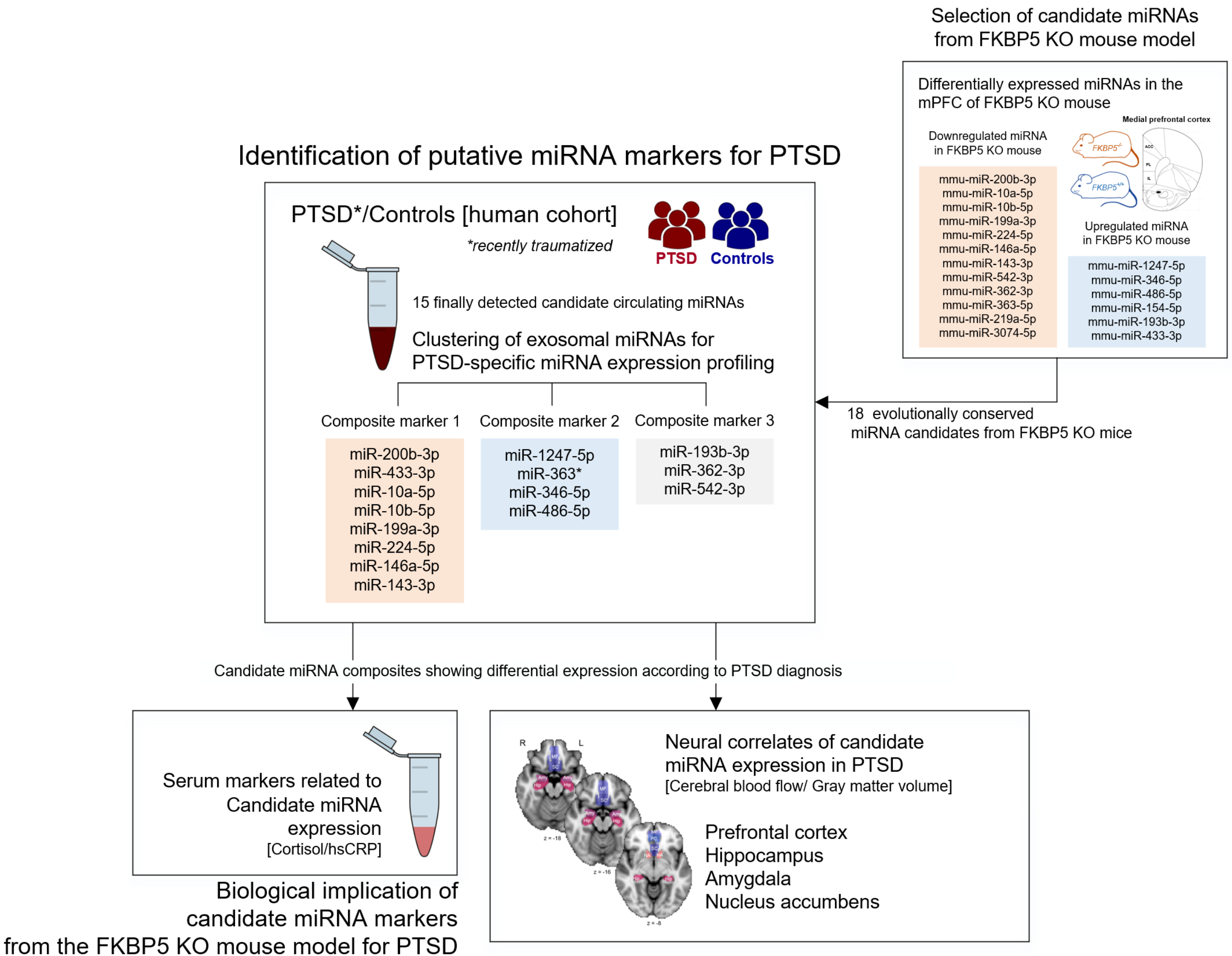 |
| --- |
| **Figure S1.** Schematic overview of the methods applied to identify miRNA markers derived from the FKBP5 KO mouse model for PTSD through a translational approach  In order to identify putative FKBP5-associated miRNA markers for PTSD, a subset of miRNAs showing differential expression in the FKBP5 KO mouse model was selected as FKBP5-associated miRNA candidates and tested in the human cohort. Expression levels of the selected FKBP5-associated candidate miRNAs were measured in the serum of recently traumatized individuals diagnosed with PTSD (n = 48) and trauma-unexposed healthy individuals (n = 47). FKBP5-associated miRNA composites that showed differential expression in PTSD were further evaluated for their relationships with serum markers including cortisol and hsCRP. Furthermore, neural correlates of PTSD-specific miRNA expression were examined by measuring cerebral blood flow and gray matter volumes within the prefrontolimbic regions of the brain. *miRNA* microRNA, *FKBP5* FK506-binding protein 5, *KO* knockout, *mPFC*, medial prefrontal cortex, *PTSD* posttraumatic stress disorder, *hsCRP* high sensitivity C-reactive protein. |

| 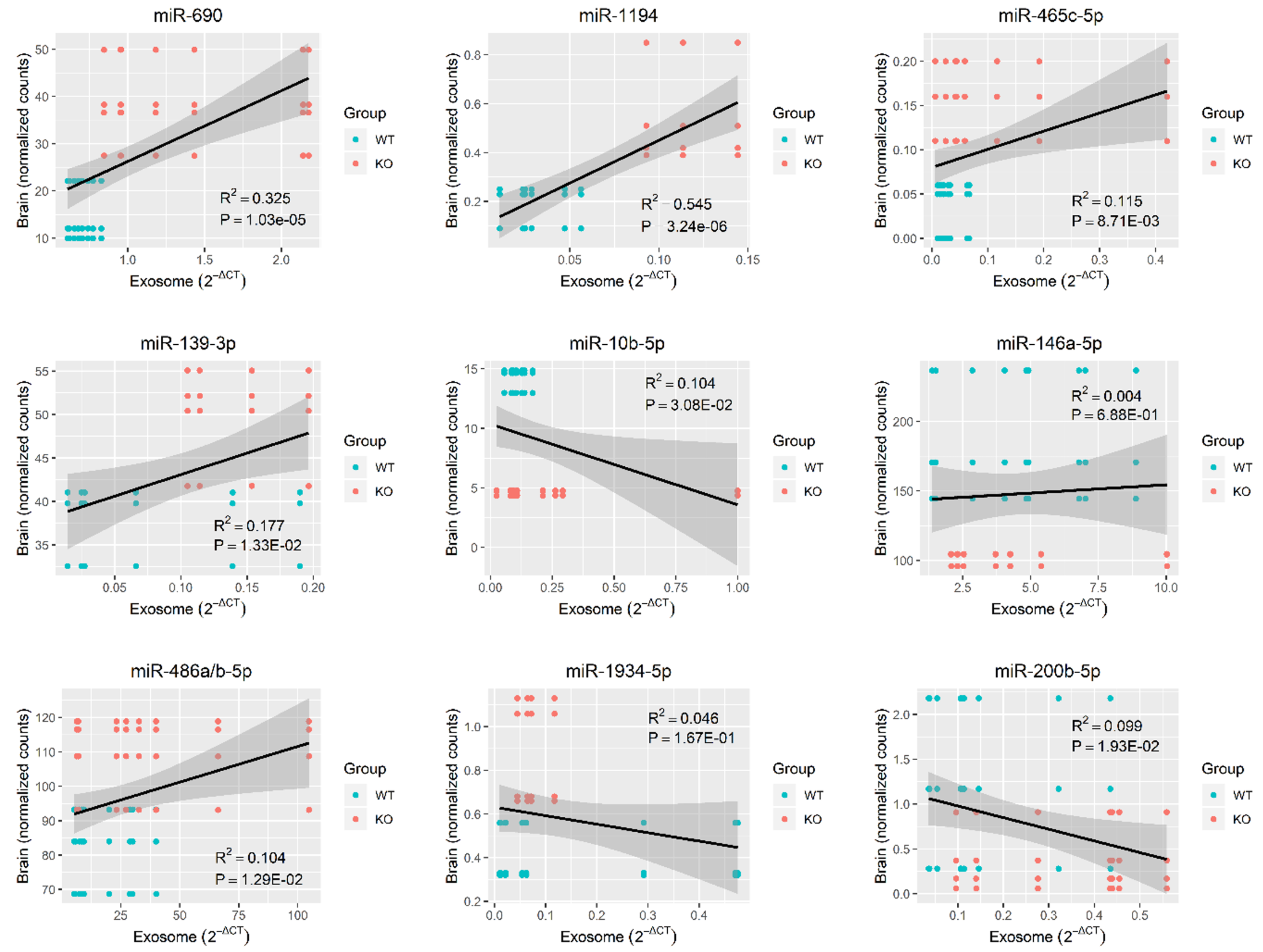 |
| --- |
| **Figure S2.** Correlations between mPFC miRNA and serum exosomal miRNA expression levels for the subset of miRNAs showing differential expression within the mPFC of FKBP5 KO mice  Linear regression analyses were performed to examine the relationships between brain sequencing and serum exosomal PCR data. The coefficients of determination (R^2^) and p-values are indicated. The black lines represent regression slopes and the dark grey areas represent 95% confidence intervals. *mPFC* medial prefrontal cortex*, miRNA* microRNA, *FKBP5* FK506-binding protein 5, *KO* knockout, *PCR,* polymerase chain reaction. |

| 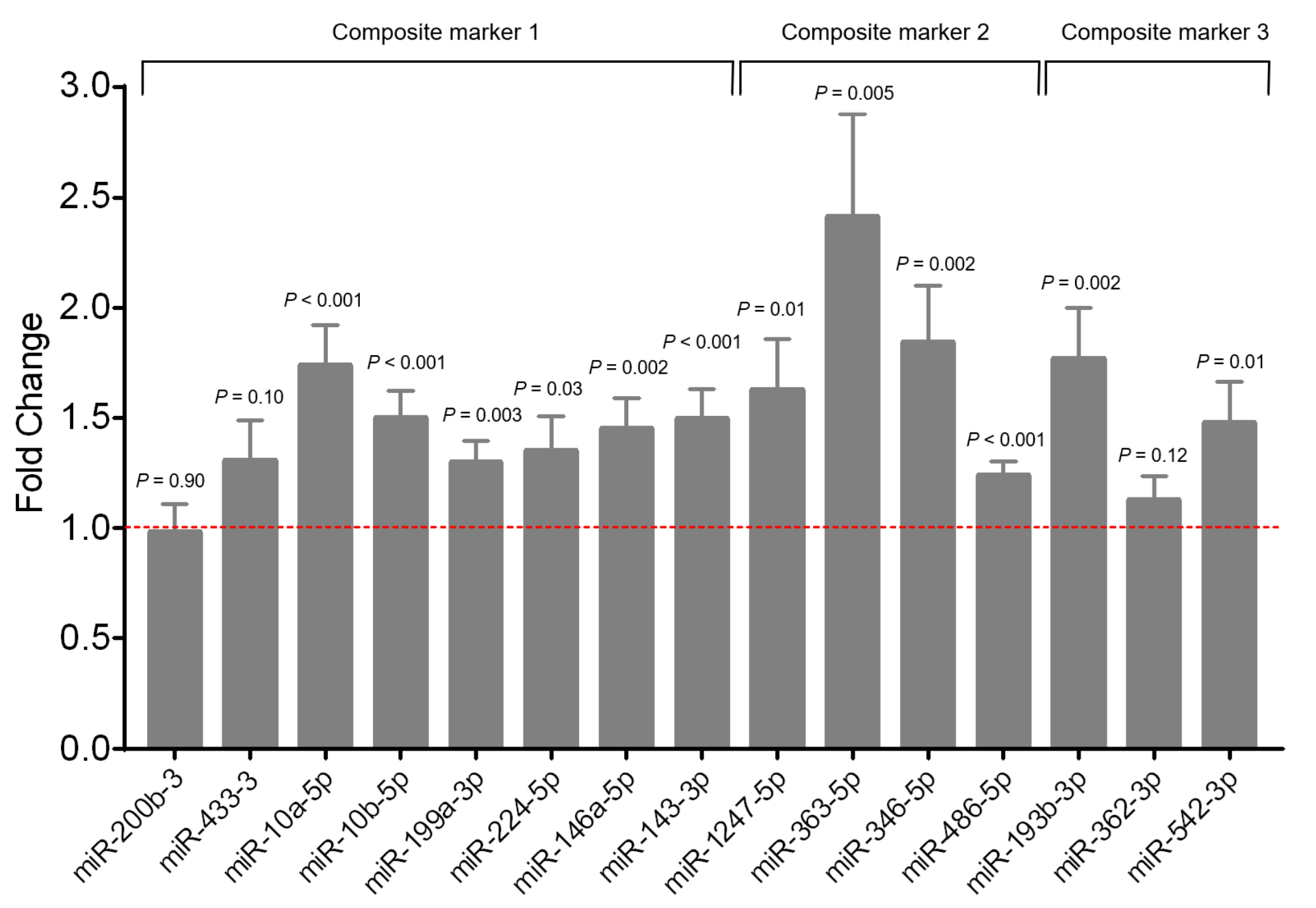 |
| --- |
| **Figure S3.** Fold changes of the PTSD group relative to the control group, All values were normalized by changes in miR-16. Error bars indicate standard errors of means. *PTSD*, posttraumatic stress disorder. |

| 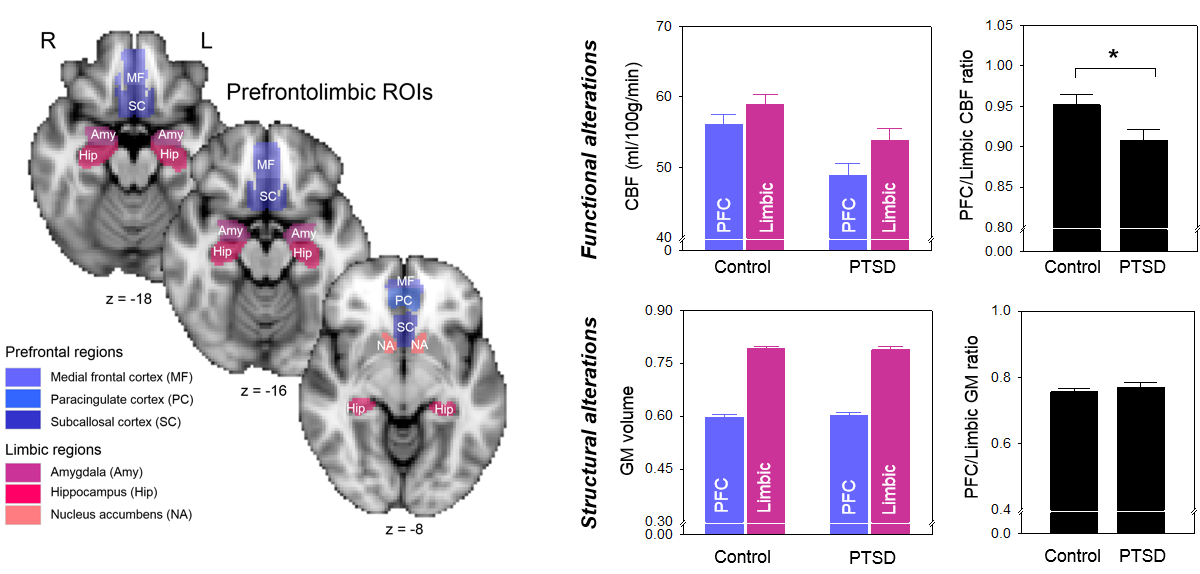 |
| --- |
| **Figure S4.** Differences in cerebral blood flow and gray matter volume in the prefrontolimbic ROIs between the PTSD and control groups.  Error bars indicate standard error of the mean. Asterisk (*) in the bar graph indicate a significant group difference between the PTSD and control groups at *P* < 0.05. *ROI* region-of-interest, *PTSD* posttraumatic stress disorder, *PFC* prefrontal cortex, *CBF* absolute cerebral blood flow, *GM* gray matter. |

| 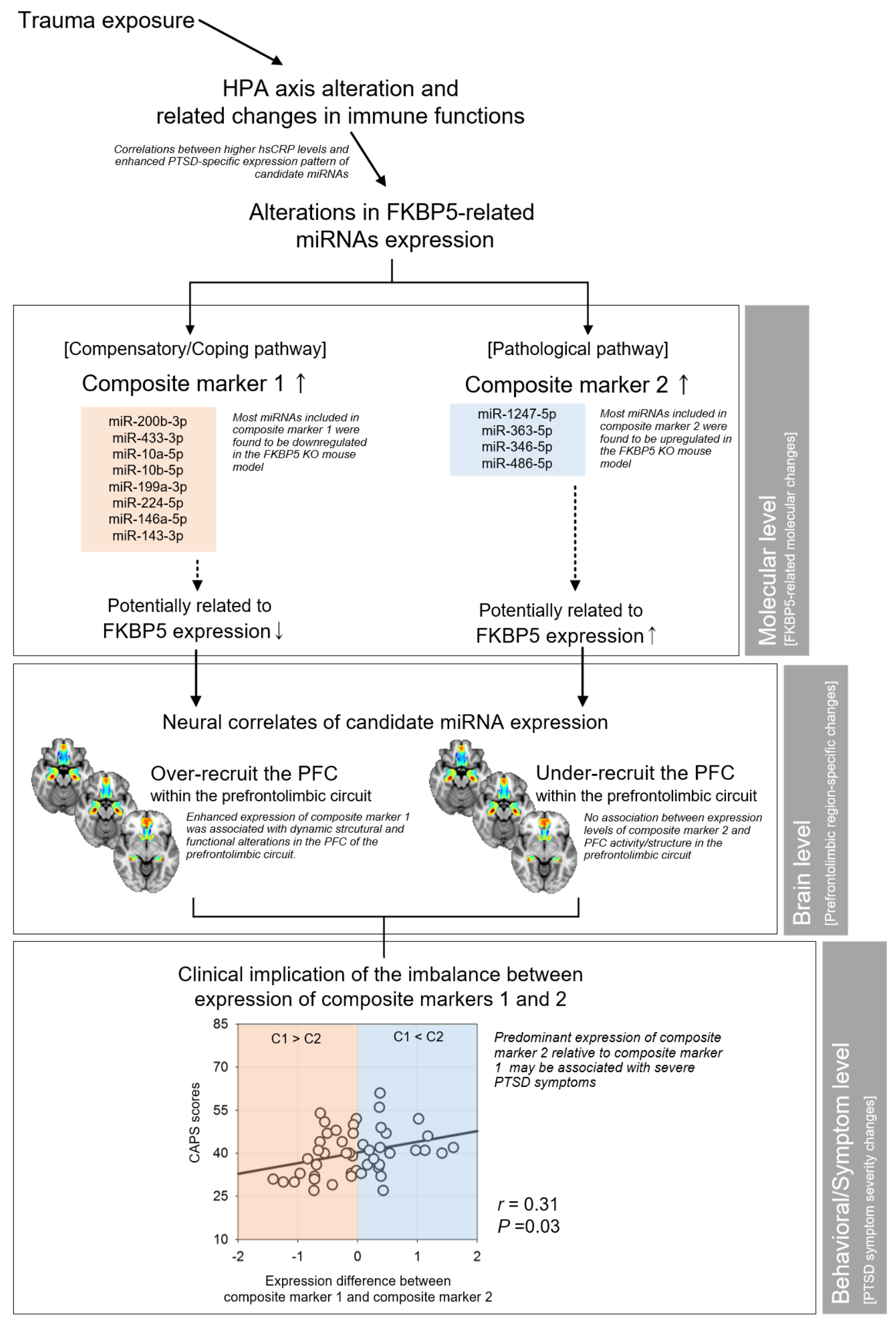 |
| --- |
| **Figure S5.** Working hypothesis of the role of expression profiles of candidate miRNAs derived from the FKBP5 KO mouse model in PTSD  In the current study, we theorized that traumatic load may alter hypothalamically regulated endocrine functions, which may influence other molecular processes such as immune systems. This may be followed by reciprocal alterations in the expression of candidate miRNAs derived from the FKBP5 KO mouse model as epigenetic regulators. Expression levels of the two subsets of candidate miRNAs (composite markers 1 and 2) were altered in recently traumatized individuals with PTSD relative to healthy individuals. The enhanced expression in composite marker 1 was associated with higher activity levels within the prefrontal regions relative to the limbic regions, which may indicate an over-activation of the prefrontal region of the brain as means to downregulate the limbic activity. Since most of the miRNAs clustered into composite marker 1 demonstrated downregulation within the prefrontal regions of the FKBP5 KO mouse model, this subset of miRNAs may play an assisting role in inhibiting the transcription of FKBP5. Therefore, we suggest that the enhanced expression of miRNAs in composite marker 1 may have a compensatory/coping mechanism that may attribute to the downregulation and/or reduced function of FKBP5. In contrast, this relationship was not observed between the expression of composite marker 2 and the relative activity of the prefrontal regions. This speculation may be partially supported by our findings at the behavioral/system level, which indicated that the imbalance between composite markers 1 and 2 may be associated with PTSD symptom severity. Specifically, the predominant expression of composite marker 2 relative to composite marker 1 was associated with more severe symptoms of PTSD. *miRNA* microRNA, *FKBP5* FK506-binding protein 5, *PTSD* posttraumatic stress disorder, *HPA* axis hypothalamus-pituitary-adrenal axis, *hsCRP* high sensitivity C-reactive protein, *KO* knockout, *PFC* prefrontal cortex, *CAPS* Clinician-Administered Posttraumatic Stress Disorder Scale for DSM-5. |

**Table S1.** Selected candidate subset of miRNAs from FKBP5 KO mouse model for the application to the human cohort of PTSD

| miRNAs | Fold changes | *P* values | Conservation in humans^1^ |
| --- | --- | --- | --- |
| *Downregulated in the mPFC of FKBP5^-/-^ mice* | | | |
| mmu-miR-146a-5p | 0.598 | < 0.001 | Yes |
| mmu-miR-10b-5p | 0.484 | 0.003 | Yes |
| mmu-miR-224-5p | 0.567 | 0.005 | Yes |
| mmu-miR-10a-5p | 0.614 | 0.01 | Yes |
| mmu-miR-200b-5p | 0.302 | 0.01 |  |
| mmu-miR-7226-3p | 0.236 | 0.01 |  |
| mmu-miR-200b-3p | 0.366 | 0.02 | Yes |
| mmu-miR-3100-5p | 0.096 | 0.02 |  |
| mmu-miR-7668-3p | 0.087 | 0.02 |  |
| mmu-miR-429-3p | 0.315 | 0.02 |  |
| mmu-miR-3074-5p | 0.587 | 0.03 | Yes |
| mmu-miR-6902-5p | 0.259 | 0.03 |  |
| mmu-miR-466i-3p | 0.325 | 0.03 |  |
| mmu-miR-143-3p | 0.751 | 0.03 | Yes |
| mmu-miR-8101 | 0.132 | 0.03 |  |
| mmu-miR-219a-5p | 0.649 | 0.03 | Yes |
| mmu-miR-7061-5p | 0.273 | 0.04 |  |
| mmu-miR-542-3p | 0.692 | 0.04 | Yes |
| mmu-miR-706 | 0.235 | 0.04 |  |
| mmu-miR-199a-3p | 0.783 | 0.04 | Yes |
| mmu-miR-199b-3p | 0.783 | 0.04 |  |
| mmu-miR-362-3p | 0.671 | 0.04 | Yes |
| mmu-miR-363-5p | 0.268 | < 0.05 | Yes |
| *Upregulated in the mPFC of FKBP5^-/-^ mice* | | | |
| mmu-miR-690 | 2.513 | < 0.001 |  |
| mmu-miR-3099-3p | 1.526 | < 0.001 |  |
| mmu-miR-1247-5p | 1.987 | 0.008 | Yes |
| mmu-miR-433-3p | 1.349 | 0.009 | Yes |
| mmu-miR-1934-5p | 2.162 | 0.009 |  |
| mmu-miR-1194 | 2.774 | 0.01 |  |
| mmu-miR-465c-5p | 3.812 | 0.01 |  |
| mmu-miR-3085-5p | 8.184 | 0.01 |  |
| mmu-miR-6906-3p | 8.217 | 0.01 |  |
| mmu-miR-486a-5p | 1.315 | 0.02 | Yes |
| mmu-miR-486b-5p | 1.315 | 0.02 |  |
| mmu-miR-154-5p | 1.560 | 0.03 | Yes |
| mmu-miR-193b-3p | 1.792 | 0.03 | Yes |
| mmu-miR-1895 | 1.509 | 0.04 |  |
| mmu-miR-6418-3p | 1.844 | 0.04 |  |
| mmu-miR-3552 | 2.169 | 0.04 |  |
| mmu-miR-139-3p | 1.298 | 0.04 |  |
| mmu-miR-346-5p | 1.298 | 0.04 | Yes |
| *miRNA* microRNA, *FKBP5* FK506-binding protein 5, *KO* knockout, *PTSD* posttraumatic stress disorder, *mPFC* medial prefrontal cortex.  ^1^Selected as candidate miRNA for the human study. | | | |

| **Table S2**. Cross-species evolutionarily conserved miRNAs as determined by sequence homology | | | | | |
| --- | --- | --- | --- | --- | --- |
| miRNA | Mouse | | Human | | Sequence homology |
|  | miRBASE | Mature sequence | miRBASE | Mature sequence |  |
| miR-200b-3p | MIMAT0000233 | UAAUACUGCCUGGUAAUGAUGA | MIMAT0000318 | UAAUACUGCCUGGUAAUGAUGA | 100% |
| miR-433-3p | MIMAT0001420 | AUCAUGAUGGGCUCCUCGGUGU | MIMAT0001627 | AUCAUGAUGGGCUCCUCGGUGU | 100% |
| miR-10a-5p | MIMAT0000648 | UACCCUGUAGAUCCGAAUUUGUG | MIMAT0000253 | UACCCUGUAGAUCCGAAUUUGUG | 100% |
| miR-10b-5p | MIMAT0000208 | UACCCUGUAGAACCGAAUUUGUG | MIMAT0000254 | UACCCUGUAGAACCGAAUUUGUG | 100% |
| miR-199a-3p | MIMAT0000230 | ACAGUAGUCUGCACAUUGGUUA | MIMAT0000232 | ACAGUAGUCUGCACAUUGGUUA | 100% |
| miR-224-5p | MIMAT0000671 | UAAGUCACUAGUGGUUCCGUU | MIMAT0000281 | UCAAGUCACUAGUGGUUCCGUUUAG | 80% |
| miR-146a-5p | MIMAT0000158 | UGAGAACUGAAUUCCAUGGGUU | MIMAT0000449 | UGAGAACUGAAUUCCAUGGGUU | 100% |
| miR-143-3p | MIMAT0000247 | UGAGAUGAAGCACUGUAGCUC | MIMAT0000435 | UGAGAUGAAGCACUGUAGCUC | 100% |
| miR-1247-5p | MIMAT0014800 | ACCCGUCCCGUUCGUCCCCGGA | MIMAT0005899 | ACCCGUCCCGUUCGUCCCCGGA | 100% |
| miR-363-5p | MIMAT0017076 | CAGGUGGAACACGAUGCAAUUU | MIMAT0003385 | CGGGUGGAUCACGAUGCAAUUU | 86.40% |
| miR-346-5p | MIMAT0000597 | UGUCUGCCCGAGUGCCUGCCUCU | MIMAT0000773 | UGUCUGCCCGCAUGCCUGCCUCU | 91.70% |
| miR-486-5p | MIMAT0003130 | UCCUGUACUGAGCUGCCCCGAG | MIMAT0002177 | UCCUGUACUGAGCUGCCCCGAG | 100% |
| miR-193b-3p | MIMAT0004859 | AACUGGCCCACAAAGUCCCGCU | MIMAT0002819 | AACUGGCCCUCAAAGUCCCGCU | 95.70% |
| miR-362-3p | MIMAT0004684 | AACACACCUGUUCAAGGAUUCA | MIMAT0004683 | AACACACCUAUUCAAGGAUUCA | 95.70% |
| miR-542-3p | MIMAT0003172 | UGUGACAGAUUGAUAACUGAAA | MIMAT0003389 | UGUGACAGAUUGAUAACUGAAA | 100% |
| *miRNA* microRNA. | | | | | |

**Table S3.** Previous literature review on miRNA candidates derived from the FKBP5 KO mouse model with a focus on their involvement in stress responses

| miRNA | Family | Species | Samples/  Regions | Model/Diseases | Expression Pattern | Target miRNA/ biological pathways suggested | References |
| --- | --- | --- | --- | --- | --- | --- | --- |
| *miRNAs clustered into composite marker 1* | | | | | | | |
| miR-200 | miR-8 | Male rats (Holtzman) | Frontal cortex | Repeated inescapable shock | Decreased | Target: CREB1, ZEB1, ZEB2  Pathway: Not specified | Smalheiser NR, Int J Neuropsychopharmacol, 2011 |
|  |  | Female rats (Rattus  norvegicus) | Frontal cortex | Acute stress  (20 min immobilization, 5 min forced swimming) | Decreased | Not specified | Yao Y, BMC Med, 2014 |
|  |  | Male rats (Wistar) | Hypothalamus | Nutritional stress (caloric restriction, high-fat diet-induced obesity) | Decreased | Not specified | Sangiao-Alvarellos S, Endocrinology, 2014 |
|  |  | Strain male C57BL/6J mice | Ventral tegmental areas | Chronic unpredictable mild stress | Decreased | Target: TNFSF13, POLE  Pathway:  · TNFSF13: Cytokine-cytokine receptor interaction, Intestinal immune network for IgA production, Rheumatoid arthritis  · POLE: Metabolic pathways, Purine metabolism, Pyrimidine metabolism, DNA replication, Nucleotide excision repair | Sun X, Biol Psychiatry, 2018 |
|  |  | Human | Prefrontal cortex | Major depressive disorder | Decreased | Not specified | Smalheiser NR, Plos one, 2012 |
|  |  | Human | Blood, PBMC | Major depressive disorder | Decreased | Not specified | Belzeaux R, Transl Psychiatry, 2012 |
|  |  | Rats | Prefrontal cortex | Corticosterone treatment | Decreased | Target: CREB, ETS  Pathway:  · CREB: Altered pro-inflammatory response, Neurogenesis, Cell differentiation, Corticosteroid synthesis, Cell survival | Dwivedi Y, Transl Psychiatry, 2015 |
|  |  | Male rats  (albino Sprague) | Amygdala | Restraint and tail shock | Increased | Not specified | Balakathiresan NS, J Psychiatr Res,  2014 |
| miR-433 | miR-433 | Male rats  (albino Sprague) | Amygdala | Restraint and tail shock | Increased | Not specified | Balakathiresan NS, J Psychiatr Res,2014 |
|  |  | Human | Blood, PBMC | Major depressive disorder | Increased | Not specified | Belzeaux R, Transl Psychiatry, 2012 |
| miR-10a | miR-10 | Male rats | Amygdala | Restraint and tail shock | Increased | Not specified | Balakathiresan NS, J Psychiatr Res, 2014 |
|  |  | Human | Prefrontal cortex | Major depressive disorder | Decreased | Target: HOXA1  Pathway: Not specified | Smalheiser NR, Plos one, 2012 |
| miR-10b | miR-10 | Rats | Hippocampus | Chronic unpredictable stress | Decreased | Target: BDNF  Pathway: Synaptic plasticity, Neuronal growth | Jiang Y, Int J Clin Exp Pathol, 2015 |
| miR-199a | miR-199 | Human | Blood, PBMC | PTSD | Decreased | Not specified | Zhou J, Plos one, 2014 |
|  |  | Human | Blood | Major depressive disorder | Increased | Not specified | Maffioletti E, J Affect Disord, 2016 |
|  |  | Human | Prefrontal cortex | Major depressive disorder | Changed | Not specified | Smalheiser NR, Plos one, 2012 |
| miR-146a | miR-146 | Human | Prefrontal cortex | Major depressive disorder | Decreased | Not specified | Smalheiser NR, Plos one, 2012 |
| miR-143 | miR-143 | Male rats (Holtzman) | Frontal cortex | Repeated inescapable shock | Decreased | Not specified | Smalheiser NR, Int J Neuropsychopharmacol, 2011 |
|  |  | Human | Blood, PBMC | PTSD | Decreased | Target: IGLL5  Pathway: Not specified | Bam M, Sci Rep, 2016 |
| *miRNAs clustered into composite marker 2* | | | | | | | |
| miR-363 | miR-363 | Male rats  (albino Sprague) | Amygdala | Restraint and tail shock | Increased | Not specified | Balakathiresan NS, J Psychiatr Res,2014 |
|  |  | Human | Blood, PBMC | Major depressive disorder | Increased | Not specified | Belzeaux R, Transl Psychiatry, 2012 |
| miR-486 | miR-486 | Human | Blood | PTSD | Decreased | Target: Transcription factor 7 (T-cell specific, HMG-box), Transcription factor 7 like 1, and  Transcription factor 7 like 2.  Pathway: Axon guidance, Wnt signaling pathway | Martin CG, Psychiatry Res, 2017 |
|  |  | Human | Blood, PBMC | PTSD | Decreased | Target: IGLL5  Pathway: Not specified | Bam M, Sci Rep, 2016 |
| *miRNAs clustered into composite marker 3* | | | | | | | |
| miR-193 | miR-193 | Adult male rats | Amygdala | Chronic stress (repeated immobilization) | Increased | Not specified | Meerson A, J Mol Neurosci ,2010 |
|  |  | Pubertal or adult male C57BL/6:129 F1 hybrid mice | Sperm | Chronic variable stress | Increased | Target: MTDH, GMEB2, YWHAZ  Pathway:  · MTDH, GMEB2: Chromatin regulation, DNA methylation | Rodgers AB, J Neurosci, 2013 |
|  |  | Human | Blood, PBMC | PTSD | Decreased | Target: IGLL5  Pathway: Not specified | Bam M, Sci Rep, 2016 |
|  |  | Male C57BL mice | Ventral tegmental areas | Chronic unpredictable mild stress | Decreased | Target: EVX10S, SYT15  Pathway: Not specified | Sun X, Biol Psychiatry, 2018 |
|  |  | Human | Dermal fibroblast | Major depressive disorder | Decreased | Target: HLDA-DPB1 DNAJC13, KCNJ2  Pathway: Not specified | Garbett KA, Biol. Psychiatry, 2015 |
| miR-362 | miR-362 | Male rats | Amygdala | Restraint and tail shock | Increased | Not specified | Balakathiresan NS, J Psychiatr Res,2014 |
| miR-542 | miR-542 | Human | Dermal fibroblasts | Major depressive disorder | Increased | Target: KIAA1324L  Pathway: Not specified | Garbett KA, Biol Psychiatry, 2015 |

*miRNA* microRNA, *FKBP5* FK506-binding protein 5, *DNA* deoxyribonucleic acid, *PTSD* posttraumatic stress disorder.

**References for Supplementary Information**

1. Weathers, F. W. *et al*. The Clinician-Administered PTSD Scale for DSM-5 (CAPS-5). Interview available from the National Center for PTSD at http://www.ptsd.va.gov (2013).

2. Desikan, R. S. *et al*. An automated labeling system for subdividing the human cerebral cortex on MRI scans into gyral based regions of interest. *Neuroimage*. **31**, 968-980 (2006).

3. Alsop, D. C. *et al*. Recommended implementation of arterial spin-labeled perfusion MRI for clinical applications: A consensus of the ISMRM perfusion study group and the European consortium for ASL in dementia. *Magn. Reson. Med.***73**, 102-116 (2015).

4. Chappell, M. A., Groves, A. R., Whitcher, B. & Woolrich, M. W. Variational Bayesian inference for a nonlinear forward model. *IEEE. Trans. Signal. Process*. **57**, 223-236 (2009).

5. Chappell, M. A. *et al*. Separation of macrovascular signal in multi-inversion time arterial spin labelling MRI. *Magn. Reson. Med.* **63**, 1357-1365 (2010).

6. Zhang Y, Brady M, Smith S. Segmentation of brain MR images through a hidden Markov random field model and the expectation-maximization algorithm. *IEEE Trans Med Imaging.* **20,** 45-57 (2001).

7. Bassett, D. S. *et al*. Hierarchical organization of human cortical networks in health and schizophrenia. *J. Neurosci.* **28,** 9239-9248 (2008).

8. Im, J. J. *et al*. Diagnostic potential of multimodal neuroimaging in posttraumatic stress disorder. *PloS One.* **12,** e0177847; 10.1371/journal.pone.0177847 (2017).

9. Lee, K. J. & Carlin, J. B. Multiple imputation for missing data: fully conditional specification versus multivariate normal imputation. *Am. J. Epidemiol*. **171,** 624-632 (2010).

10. Westfall, P. H. & Young, S. S. Resampling-based multiple testing: examples and methods for p-value adjustment. (John Wiley & Sons, 1993).

11. Schell, R. M. *et al.* Cerebral blood flow and metabolism during cardiopulmonary bypass. Anesth Analg. **76,** 849-865 (1993).

12. Bjorklund, G. *et al.* Cerebral hypoperfusion in autism spectrum disorder. Acta Neurobiol Exp (Wars). **78,** 21-29 (2018).

13. Belzeaux, R. *et al.* Responder and nonresponder patients exhibit different peripheral transcriptional signatures during major depressive episode. *Transl. Psychiatry*. **2,** e185; 10.1038/tp.2012.112 (2012).

14. Smalheiser, N. R. *et al*. MicroRNA expression is down-regulated and reorganized in prefrontal cortex of depressed suicide subjects. *PloS One*. **7,** e33201; 10.1371/journal.pone.0033201 (2012).

15. Smalheiser, N. R. *et al*. MicroRNA expression in rat brain exposed to repeated inescapable shock: differential alterations in learned helplessness vs. non-learned helplessness. *Int. J. Neuropsychopharmacol*. **14,** 1315-1325 (2011).

16. Sun, X. *et al*. microRNA and mRNA profiles in ventral tegmental area relevant to stress-induced depression and resilience. *Prog. Neuropsychopharmacol. Biol. Psychiatry.* **86,** 150-165 (2018).

17. Balakathiresan, N. S. *et al*. Serum and amygdala microRNA signatures of posttraumatic stress: fear correlation and biomarker potential. *J. Psychiatr. Res*. **57,** 65-73 (2014).

18. Dwivedi, Y. *et al*. Chronic corticosterone-mediated dysregulation of microRNA network in prefrontal cortex of rats: relevance to depression pathophysiology. *Transl. Psychiatry.* **5,** e682; 10.1038/tp.2015.175 (2015).

19. Yao, Y. *et al*. Ancestral exposure to stress epigenetically programs preterm birth risk and adverse maternal and newborn outcomes*. BMC. Med*. **12,** 121; 10.1186/s12916-014-0121-6 (2014).

20. Sangiao-Alvarellos, S. *et al.* Perturbation of hypothalamic microRNA expression patterns in male rats after metabolic distress: impact of obesity and conditions of negative energy balance. *Endocrinology.* **155,** 1838-1850 (2014).

21. Jiang, Y. & Zhu, J. Effects of sleep deprivation on behaviors and abnormal hippocampal BDNF/miR-10B expression in rats with chronic stress depression. *Int. J. Clin. Exp. Pathol.* **8,** 586-593 (2015).

22. Zhou, J. *et al*. Dysregulation in microRNA expression is associated with alterations in immune functions in combat veterans with post-traumatic stress disorder. *PLoS One.* **9,** e94075; 10.1371/journal.pone.0094075 (2014).

23. Maffioletti, E. *et al*. Peripheral whole blood microRNA alterations in major depression and bipolar disorder. *J. Affect. Disord.* **200**, 250-258 (2016).

24. Bam, M. *et al*. Dysregulated immune system networks in war veterans with PTSD is an outcome of altered miRNA expression and DNA methylation. *Sci. Rep.* **6,** 31209; 10.1038/srep31209 (2016).

25. Martin, C. G. *et al*. Circulating miRNA associated with posttraumatic stress disorder in a cohort of military combat veterans. *Psychiatry. Res*. **251,** 261-265 (2017).

26. Hollins, S. L. & Cairns, M. J. MicroRNA: small RNA mediators of the brains genomic response to environmental stress. *Prog. Neurobiol.* **143,** 61-81 (2016).

27. Garbett, K. A. *et al*. Coordinated messenger RNA/microRNA changes in fibroblasts of patients with major depression. *Biol. Psychiatry.* **77,** 256-265 (2015).
